# A juvenile ALS‐like phenotype dramatically improved after high‐dose riboflavin treatment

**DOI:** 10.1002/acn3.50977

**Published:** 2020-02-05

**Authors:** Christophe Carreau, Timothée Lenglet, Isabelle Mosnier, Ghizlene Lahlou, Guillaume Fargeot, Nicolas Weiss, Sophie Demeret, François Salachas, Alice Veauville‐Merllié, Cécile Acquaviva, Yann Nadjar

**Affiliations:** ^1^ Department of Neurology Groupe Hospitalier Pitié‐Salpêtrière (AP‐HP) 47‐83 Boulevard de l'Hôpital 75013 Paris France; ^2^ Department of Neurophysiology Groupe Hospitalier Pitié‐Salpêtrière (AP‐HP) 47‐83 Boulevard de l'Hôpital 75013 Paris France; ^3^ Department of Otology, Auditory Implants and Skull Base Surgery Groupe Hospitalier Pitié‐Salpêtrière (AP‐HP) Paris France; ^4^ Department of Inborn Errors of Metabolism and Neonatal Screening Center of Biology and Pathology Est CHU Lyon Bron France

## Abstract

Riboflavin transporter deficiency (RTD) was recently characterized as a cause of genetic recessive childhood‐onset motor neuron disease (MND) with hearing loss, formerly described as Brown‐Vialetto‐Van‐Lear syndrome. We describe a 18‐year‐old woman with probable RTD mimicking juvenile Amyotrophic Lateral Sclerosis (ALS) who presented with an inaugural respiratory failure and moderate distal four limbs weakness. Only one heterozygous SLC52A3 mutation was detected, but presence of a sub‐clinical auditory neuropathy and dramatic improvement under high dose riboflavin argued for a RTD. As RTD probably has a larger phenotypic spectrum than expected, a high dose riboflavin trial should be discussed in young‐onset MND.

## Introduction

Brown‐Vialetto‐Van‐Laere (BVVL) syndrome was originally described as a recessive hereditary pediatric onset disease associating a Motor neuron disease (MND), with characteristic pontobulbar palsy, and hearing loss.^1^ Prognosis is poor due to a frequent occurrence of a respiratory failure. Recently, genetic basis of BVVL was partly disclosed, with some patients harboring bi‐allelic pathogenic variants either in *SLC52A2* or *SLC52A3* gene that were shown to act as riboflavin transporters.^2^ Riboflavin transporter deficiency (RTD) patients may present some biochemical signs that can guide toward the diagnosis: abnormal acylcarnitine profile and low riboflavin level in blood.^3^ Some RTD patients clearly improved with high‐dose riboflavin treatment.^4^ Here we describe the case of an 18‐year‐old woman with probable RTD mimicking juvenile Amyotrophic lateral sclerosis (ALS) who presented with an inaugural respiratory failure and dramatically improved with riboflavin.

## Case Report

An 18‐year‐old woman was hospitalized in intensive care for respiratory distress with severe hypercapnia suggesting diaphragmatic dysfunction (PaO2 48 mmHg, PaCO2 74 mmHg, HCO_3_‐ 41 mmol/L), the day after her arrival by plane in France from a sub‐Saharian region. The breathing difficulties had started one year before by exertional dyspnea and progressed until hospitalization. There was no additional medical history, no familial history, and no parental consanguinity. The patient rapidly needed mechanical ventilation because of ineffective Non‐invasive ventilation (NIV).

**Figure 1:**
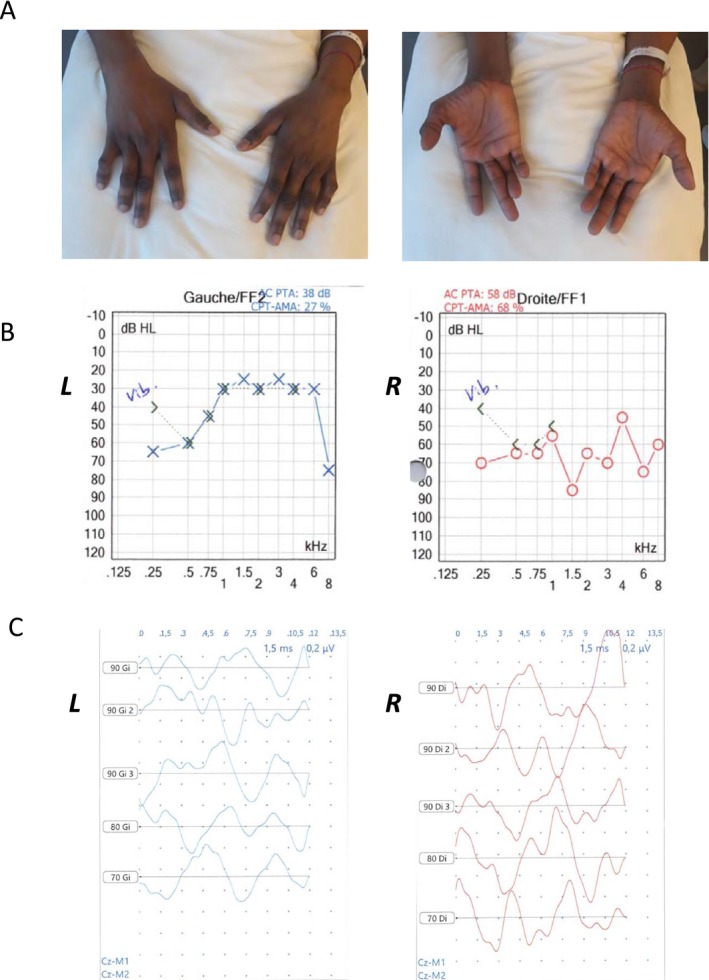
(A) Amyotrophy in both hands. B and C (L: Left, R: Right): Pure‐tone audiometry showing bilateral hearing loss with an air‐conduction average of 62 and 43 dB for the right and the left ear, respectively (B); auditory brainstem response showing the absence of compound action potentials (C).

The clinical examination showed a weakness of bilateral interossei muscles at 3/5 MRC score in upper limbs and of bilateral psoas‐iliac and tibialis anterior muscles in lower limbs at 4/5. Amyotrophy was observed in distal four limbs (Fig. 1A), not associated with fasciculations. Reflexes were all present. There was no pyramidal syndrome. There was no sensory disorder and no cranial nerve involvement. Her clinical examination remained stable during her 6 weeks stay in intensive care.

Motor nerve conduction study found compound motor action potentials of decreased amplitude in four limbs with normal conduction velocities (Table S1); there was no decrement on repetitive nerve stimulation; sensory conduction was normal; needle examination showed spontaneous activity (i.e., fibrillation potentials and/or positive sharp waves) and neurogenic recruitment in bilateral interossei and tibialis anterior muscles arguing for a MND; fasciculation potentials were absent (Table S2).

All biological investigations were normal, notably auto‐immune, infectious and metabolic markers including CSF examination, acylcarnitine profile, and riboflavin in blood. Brain and spinal cord MRIs were normal, as the thoraco‐abdominal CT scan. The muscle biopsy showed only abnormalities related to a pure neurogenic disease.

An Ig IV treatment was given at 2 g/kg the day after her hospitalization to treat potential auto‐immune disease (Guillain‐Barré syndrome), with no observed efficiency. After 1 month with failure to wean mechanical ventilation, tracheostomy was decided, and a high dose per os riboflavin trial (15 mg/kg) was initiated. Her clinical condition then very slowly improved – tracheostomy removal was possible 7 months after treatment onset (M7) with discontinuation of diurnal ventilation but need for nocturnal NIV; at M16 she only used nocturnal NIV three nights a week; at M20 she could totally stop NIV with no sign of hypoventilation when evaluated after 1 month (notably normal pCO_2_ after night sleep). Vital Capacity was measured at 1690mL at M4, increased at 2730 mL (+67%) at M15. Limb weakness did not obviously improve whereas motor nerve conduction study showed a slightly improvement after 1 year, mostly in lower limbs (Table S1). She was independent for all daily activities.

Using a next generation sequencing (NGS) approach targeting exons and exon–intron boundaries, genetic investigations for RTD revealed a never‐reported likely pathogenic heterozygous missense variant in *SLC52A3* gene c.113G > C (see Table 1 for in silico data), classified as a variant of unknown significance type 4 (VUS 4). Gene dosage anomalies have been excluded as our bioinformatic pipeline allows for copy number variation (CNV) detection. Familial segregation study was not possible but both parents were alive and healthy. No mutation was identified in ALS genes (NGS for *FUS*, *TARDP*, and *SOD1*, and hexanucleotide repeat analysis for *C9ORF72* were performed). To assess further the RTD diagnosis, as only one likely pathogenic variant was found, auditory and visual explorations were performed whereas there was no patient’s complaint. Pure‐tone audiometry test showed a bilateral sensorineural hearing loss with an air‐conduction average of 62 and 43 dB for the right and the left ear, respectively (Fig. 1B–C). The absence of compound action potentials on auditory brainstem response and the presence of otoacoustic emissions confirmed the diagnosis of auditory neuropathy. Evoked visual potentials were also abnormal with low and delayed potentials.

**Table 1:** Frequency, in silico prediction and conservation for the variant c.113G>C (p.Trp38Ser).

Frequency			In silico prediction				Conservation
gnomAD	ESP	ExAc	Polyphen2	Mutation taster	SIFT	GVGD	
Absent	Absent	Absent	Probably damaging (score = 1.000)	Pathogen	Deleterious (score = 0)	C65	Very conserved among species and in the two other cytoplasmic transporters for riboflavin in human

## Discussion

We report here a very unusual presentation of a probable RTD: our patient did not suffer from overt hearing loss, and had a late‐onset MND, with no signs of bulbar or pontine localizations. Usually, in RTD: (1) age at first manifestations occurs earlier; (2) the neuro‐sensorial disorder is a key feature of the disease, and very often the first manifestation; (3) and finally the motor neuron phenotype is characterized by particular localizations, bulbar but also pontine with lower and upper facial weakness. In addition, blood acylcarnitine profile and riboflavin were normal in our patient; however, there is increasing evidence that these biochemical abnormalities are often absent in adults with RTD.^5^ First diagnostic hypothesis was juvenile ALS, due to inaugural diaphragmatic dysfunction associated with a diffuse pure motor neurogenic disorder.

Despite no specific clinical or biological argument for RTD,^4^ riboflavin trial was decided considering unusual features for ALS – the very young age of the patient, the symmetric pattern of weakness, and the absence of fasciculations. RTD suspicion must lead to specifically treat early, without waiting for genetic confirmation.^6^

We observed and clearly documented a dramatic and durable improvement in diaphragmatic function in several months with this treatment. Moderate weakness in distal four limbs did not clearly improve under riboflavin, probably due to its pre‐existence to the diaphragmatic disorder, with less probability of reversibility when offering the specific treatment. This clinical evolution greatly oriented toward RTD diagnosis, as this is an unexpected course in other causes of motor neuron diseases, especially juvenile ALS. However, results of genetic testing were equivocal as only one likely pathogenic variant was detected, without identification of a second variant. This second mutation may be difficult to detect (deep intronic regions and promotor were not screened), as in several reported RTD cases, interestingly always with a late onset (Table S3)^7^, ^8^, ^9^. It could be that this second mutation is very mild, if located for example in regulatory parts of the gene, explaining why a single pathogenic variant status is associated with less severe forms of RTD. Concerning the variant that was found (c.113G > C) in our patient, its nature as a missense variant could possibly lead to a partial and not total loss of function of the transporter. Mutations found in RTD are various, including truncating variants potentially more severe than point mutations; however, in our experience and what is known from literature those supposed “severe” mutations are not clearly more frequent in clinically severe RTD with pediatric onset than late‐onset RTD.^5^

To assess the diagnosis, we performed visual and auditory evoked potentials, even without patient's complaint, and an auditory neuropathy was demonstrated, as described in RTD^10^, strongly arguing for this diagnosis. We also found a subclinical visual impairment, probably an optic atrophy which is also described in RTD but more often with *SLC52A2* variants than *SLC52A3*. Our report demonstrates that subclinical abnormal evoked potentials can be interesting diagnostic markers of RTD, especially in adults where other markers, biochemical but also genetic, can lack diagnostic sensitivity.

In conclusion, we show here that RTD probably has a larger phenotypic spectrum than expected, potentially mimicking ALS as here. Due to the known difficulties to establish this diagnosis, a high‐dose riboflavin trial should be discussed in young‐onset MND, even without bulbar and/or pontine localization, and even without evidence of hearing loss. Evoked potentials should also be considered in the diagnostic process.

## Conflict of Interest

CC, TL, IM, GL, GF, NW, SD, FS, AV, and CA have nothing to disclose. YN received speech honoraria from Actelion and Orphan Europe; and received travel funding from Actelion, Shire and Genzyme.

## Author Contribution

CC and YN contributed equally in the conception of the manuscript, the acquisition of the data, and drafting of a significant portion of the manuscript and the figure. IM, TL, GL, GF, NW, SD, FS, AV, and CA contributed in drafting and editing a significant portion of the manuscript. All authors approved the final manuscript.

## Supporting information


**Table S1.** Electrophysiological features at baseline and one year after treatment. Bold values are under the normal laboratory value and underlined if significantly improved under treatment
**Table S2.** Electrophysiological pattern in detection show a symmetrical distal active denervation, without bulbar involvement. There was no myotonic salve nor fasciculation. 0: none, +: slight, ++: moderate, +++: important
**Table S3.** List of published RTD cases with a single mutation found in the heterozygous state
