# Supplementary material for: A juvenile ALS‐like phenotype dramatically improved after high‐dose riboflavin treatment
Source: Ann Clin Transl Neurol. 2020 Feb 5;7(2):250–3. doi: 10.1002/acn3.50977 (PMC7034506; doi:10.1002/acn3.50977)
Supplement: Supplementary file 1 — Table S1. Electrophysiological features at baseline and one year after treatment. Bold values are under the normal laboratory value and underlined if significantly improved under treatment Table S2. Electrophysiological pattern in detection show a symmetrical distal active denervation, without bulbar involvement. There was no myotonic salve nor fasciculation. 0: none, +: slight, ++: moderate, +++: important Table S3. List of published RTD cases with a single mutation found in the heterozygous state [file ACN3-7-250-s001.docx]

ADDITIONAL DATA

**Table 1 supp.** Electrophysiological features at baseline and one year after treatment. Bold values are under the normal laboratory value and underlined if significantly improved under treatment.

| **NCS data** | **Patient at diagnosis** | | **Patient after one year of treatment** | | **Normal values** |
| --- | --- | --- | --- | --- | --- |
| **NR** | Left | Right | Left | Right | NR |
|  | **MOTOR CONDUCTION** | | | | |
|  | **MEDIAN NERVE** | | | | |
| **Amplitude (mV)** | **0.69** | **0.76** | **0.06** | **0.27** | >6 |
| **Velocity (m/s)** | **38** | 48 | ND | **40** | >48 |
|  | **ULNAR NERVE** | | | | |
| **Amplitude (mV)** | **4.87** | **5.90** | 6.51 | **5.91** | >6 |
| **Velocity (m/s)** | 52 | 56 | 55 | 61 | >48 |
|  | **PERONEAL NERVE** | | | | |
| **Amplitude (mV)** | **1.99** | 2.99 | 4.60 | 4.21 | >3 |
| **Velocity (m/s)** | 43 | 50 | 58 | 62 | >42 |
|  | **POSTERIOR TIBIAL NERVE** | | | | |
| **Amplitude (mV)** | **1.33** | **0.80** | **2.15** | **1.46** | >6 |
| **Velocity (m/s)** | ND | ND | ND | ND | >42 |
|  | **SENSORY CONDUCTION** | | | | |
|  | **MEDIAN NERVE** | | | | |
| **Amplitude (mV)** | 40.5 | 24.7 | 69.3 | 187.1 | >15 |
| **Velocity (m/s)** | 49 | 45 | 46 | 44 | >45 |
|  | **MUSCULOCUTANEOUS NERVE** | | | | |
| **Amplitude (mV)** | 13.7 | 13.1 | 17.1 | 21 | >10 |
| **Velocity (m/s)** | 39 | 39 | ND | ND | >40 |

NA: Not Available. Abnormal values are in bold; No: no obtained potential.

**Table 2 supp.** Electrophysiological pattern in detection show a symmetrical distal active denervation, without bulbar involvement. There was no myotonic salve nor fasciculation. 0: none, +: slight, ++: moderate, +++: important.

| **Muscle** | **Spontaneous activity**  **(positive sharp waves and/or fibrillation potential)** |
| --- | --- |
|  |  |
| Genioglossus | 0 |
| Mentalis | 0 |
| Orbicularis oris | 0 |
| Trapezius | 0 |
| Deltoid | 0 |
| Extensor carpi radialis | + |
| First dorsal interossei | +++ |
| Vastus lateralis | 0 |
| Tibialis anterior | +++ |

**Table 3 supp.** List of published RTD cases with a single mutation found in the heterozygous state.

| **Gene affected** | **Study** | **Patient identification** | **Genetics** |
| --- | --- | --- | --- |
| *SLCA52A3* | Cicoccella and al. 2012 [1] | Case 5 | c.1296C>A |
|  | Dezfouli and al. 2012 [2] | Case 2 | c.C659A |
|  | Dezfouli and al. 2012 [2] | Case 3 | c.G1124A |
|  | Manole and al. 2017 [3] | AP3 | c.1371C>G; p.Phe457Leu |
|  | Manole and al. 2017 [3] | AP4 | c.37G>A; p.Gly13Arg |
|  | Manole and al. 2017 [3] | AP5 | c.37G>A; p.Gly13Arg |
|  | Manole and al. 2017 [3] | AP7 | c.374C>A; **p.Thr125Asn** |
|  | Manole and al. 2017 [3] | AP8 | c.403A>G; **p.Thr135Ala** |
|  | Manole and al. 2017 [3] | AP9 | c.58A>C; **p.Ile20Leu** |
| *SLCA52A3* and *SLC52A2* | Allison and al. 2017 [4] | Case 1 | c.353C>A / - (*SLC52A2*); c.106G>A / - (*SLC52A3*) |

1. Ciccolella, M. et al. Brown-Vialetto-van Laere and Fazio-Londe overlap syndromes: a clinical, biochemical and genetic study. Neuromuscul. Disord. 22, 1075–1082 (2012).
2. Dezfouli, M. A., Yadegari, S., Nafissi, S. & Elahi, E. Four novel C20orf54 mutations identified in Brown-Vialetto-Van Laere syndrome patients. J. Hum. Genet. 57, 613–617 (2012).
3. Manole A, Jaunmuktane Z, Hargreaves I, *et al.* Clinical, pathological and functional characterization of riboflavin-responsive neuropathy. *Brain* 2017;140:2820–37.
4. Allison, T., Roncero, I., Forsyth, R., Coffman, K. & Pichon, J.-B. L. Brown-Vialetto-Van Laere Syndrome as a Mimic of Neuroimmune Disorders: 3 Cases From the Clinic and Review of the Literature. J. Child Neurol. 32, 528–532 (2017).
